# Indexes of jaw muscle function in asymptomatic individuals with different occlusal features

**DOI:** 10.1002/cre2.140

**Published:** 2018-11-28

**Authors:** Francesca Vozzi, Lorenzo Favero, Redento Peretta, Luca Guarda‐Nardini, Francesco Cocilovo, Daniele Manfredini

**Affiliations:** ^1^ School of Dentistry University of Padova Italy; ^2^ Private Practice of Orthodontics Italy; ^3^ Department of Dentistry and Maxillofacial Surgery Hospital of Treviso Italy; ^4^ School of Dentistry University of Siena Italy

**Keywords:** masticatory muscles, muscle function indexes, surface electromyography

## Abstract

This study aims to assess the correlation between indexes of jaw muscle function and dento‐skeletal morphology. A sample of 35 temporomandibular disorders‐free healthy individuals (10 males, mean age 26.7 ± 9.8 years) underwent surface electromyographic (sEMG) assessment of bilateral masseter and temporalis muscles, to evaluate sEMG activity during maximum voluntary clenching (MVC) with a dedicated device (Easymyo®, T.F.R. Technology, Udine, Italy). Four outcome parameters were assessed for each individual: MCV on cotton rolls; MVC on teeth; chewing on right and left sides; clench/relax test. Electromyographic recordings were assessed based on five standardized indexes of muscle function, to evaluate the degree of muscle asymmetry during static and dynamic function (i.e., percentage overlapping coefficient [POC], Impact, Asymmetry, Activation, and Torque). For each individual, the presence of a number of occlusal and skeletal features was assessed: asymmetry of molar class; deviated incisor midline; deep bite; open bite; and crossbite. Skeletal class and vertical dimension of occlusion were also evaluated. Based on normality distribution of data, *t* test and analysis of variance, when needed, were used to compare muscle function indexes between individuals with and without the different dento‐skeletal features. None of the muscle function indexes (POC, Impact, Asymmetry, Activation, and Torque) was significantly different between individuals with or without the various dental and skeletal features. Gender differences were also not significant (*p* > 0.05). Despite some minor differences were observed, none of them was significant. Thus, the interaction between form and function is too complex for hypothesizing a simple one‐to‐one relationship between interarch tooth relationship and muscle function patterns.

## 1 INTRODUCTION

For years, the potential usefulness of surface electromyography (sEMG) in dentistry has been a much‐debated issue. On one hand, from a theoretical viewpoint, it allows recording amplitude and features of electric potentials generated during muscle contractions; on the other hand, its use has been mainly suggested in the field of temporomandibular disorders (TMD), as an instrument that may detect the “ideal” interarch relationship for a balanced muscle function. Unfortunately, for the advocates of this approach, the literature has repeatedly shown that in the absence of normality data, the use of sEMG in the field of TMD is a commercially driven strategy that does not actually improve the quality of cares (Reid & Greene, 2013). In particular, there is an absence of differences between individuals with and without TMD pain, which makes the adoption of sEMG‐based recommendations to restore occlusion insensate from a biological viewpoint (Manfredini et al., 2011; Manfredini et al., 2011).

Within these premises, the physiology of jaw muscle function is yet to elucidate, and some strategies to standardize the adoption of sEMG in dentistry have been proposed (Klasser & Okeson, 2006; Manfredini, Castroflorio, Perinetti, & Guarda‐Nardini, 2012; Manfredini, Cocilovo, et al., 2011; Manfredini, Cocilovo, Stellini, Favero, & Guarda‐Nardini, 2013). For instance, an interesting issue is the relationship between occlusal and skeletal morphology and jaw muscle activity (Ferrario, Tartaglia, Galletta, Grassi, & Sforza, 2006). Some works suggested that jaw muscles are influenced by dento‐skeletal features (Bakke, 1993; Bakke, Micheler, & Moller, 1992; Ferrario, Sforza, Colombo, & Ciusa, 2000; Moreno, Cattaneo, Spadaro, & Giannoni, 2008) and pointed out the need to assess the physiology of sEMG features with respect to the different occlusal patterns (Ferrario et al., 2006; Manfredini et al., 2013; Okeson, 1985).

Early works by Ferrario et al. introduced the concept of muscular steadiness of dental occlusion, which means the amount of muscle activity depending on dental occlusion (Ferrario et al., 2000). A protocol for standardizing sEMG recordings at the intraindividual level as well as indexes for evaluating muscle symmetry was thus proposed (Ferrario et al., 2000). In short, maximum voluntary contraction (MVC) on teeth is expressed as a percentage of MVC on cotton rolls, thus reflecting the amount of muscle activity which depends on the proprioception afferent from occlusal contacts. The basic concept underlying this standardization of sEMG signals is that it limits the shortcomings due to the several technical and biological factors that make interpretation of sEMG findings complex. When the value of standardized activity is within an acceptable range, the occlusion is considered muscularly steady (Ferrario et al., 2000).

Notwithstanding this theoretical framework, the proposed indexes have never been used extensively to record parameters of normal function in healthy individuals, thus further limiting any attempt to use sEMG as a diagnostic tool in dentistry. Considering these premises, the aim of this study was to assess the standardized sEMG activity and functional indexes of jaw muscles in asymptomatic subjects with different occlusal features, to test the study hypothesis that differences should exist between individuals having or not having certain occlusal traits.

## 2 MATERIALS AND METHODS

Thirty‐five TMD‐free healthy individuals (10 males, mean age 26.7 ± 9.8 years; range 24–42) took part to the study. The subjects enrolled in the protocol were not undergoing orthodontic treatment and were free of any orofacial pains or other oral or systemic disease that could influence motor functioning.

For all subjects, a series of dental and skeletal features were assessed: molar Angle class, symmetry between right and left side, presence/absence of open bite, deep bite, crossbite, and deviation of incisor midline. Intraoral photographs and tooth impressions were also taken to assess the presence of the dental features under investigation.

Each subject also underwent recordings of jaw muscles' activity with the use of a dedicated device (Easymyo®) and software (DAQ [Dental Afference Quantifier]®, T.F.R. Technology, Udine, Italy) for the standardization of sEMG activity.

All recording procedures were performed with the patient sitting with a straight back, with the backrest in vertical position and feet resting on the ground. Before electrode positioning, skin was cleaned with sterile gauze soaked in denatured alcohol. Then, the recording electrodes (Duodrode Electrodes, Myotronics, Kent, WA, USA) were placed bilaterally on the anterior temporalis and masseter muscles (four recording channels). The reference electrode was placed in the middle of the forehead, due to the low amount of sEMG interferences coming from that area.

Four recordings tasks were performed by each individual.

- *Clench on cotton rolls*—the patient was asked to perform maximum voluntary clench on cotton rolls positioned at the first molar level and keep the task for 5 s.
- *Clench on teeth*—the patient was asked to perform MVC on teeth, without any cotton rolls between the dental arches, and keep the position for 5 s.
- *Chewing*—the patient was asked to chew a gum on the left and then the right side in two different tasks of 15 s each.
- *Clench‐relax test*—the patient was asked to tap teeth rhythmically for 15 s. The rationale for this test is to identify transient occlusal disturbances that are normally compensated during prolonged MVC tests.

Each test was repeated three times per each individual, and the median value of the three attempts was considered for statistical analysis. Muscle performance was also compared between sexes.

The DAQ software generated indexes of muscle function for the different conditions under investigation (all indexes are expressed as percentage values of MVC on cotton rolls; De Felício, Sidequersky, Tartaglia, & Sforza, 2009):

- POC (percentage overlapping coefficient)—it analyzes left and right homologous muscles asymmetry during static and dynamic tasks. For instance, if muscles contract with perfect symmetry, a POC up to 100% is to be expected. It is possible to compute this coefficient for different paired muscles: masseter muscles POC (MR/ML); temporalis muscles POC (TR/TL); and mean POC (MR + TR)/(ML + TL).
- Asymmetry—this is an asymmetry index that compares right muscles activity with left muscles activity. Positive values indicate a prevalence of the right muscles activity, whereasile negative values state a prevalence of the left ones (ASY = [TR + MR] − [TL + ML]).
- Activation—it compares the influence of dental contact on masseter and temporalis muscles activity: It quantifies the different activity between temporalis and masseters (ACT = [MR + MS] − [TR + TS]). Usually, a higher masseter activity means that there is a good interarch relationship, whereas a prevalence of temporalis contraction indicates an effort to get a coincidence between upper and lower arch. Positive values are associated with higher masseter muscles contraction, instead, negative values are related to higher temporalis activity.
- Torque—it is a torque index that assesses the presence of a laterodeviating effect on the mandible during the test given by unbalanced TR and ML and TL and MR couples. It compares the activity of the paired muscles (TC = [TR + ML] − [TR + ML]). A prevalence of right or left temporal muscle activity results in a torque that determines, respectively, a jaw deviation on the right or on the left. Positive values are related with a higher right temporal muscle contraction, whereas negative values are associated with a higher left temporal muscle activity.
- Impact—it measures muscle activation in comparison with MVC on cotton rolls. This index is the key to check the validity of all the previous indexes: notwithstanding possible low muscles performance as well as asymmetry of function, some subjects can find themselves in a condition of balance if the IMP has a value of 80–120% (De Felício et al., 2009).

A condition in which all these indexes are included within a purported physiological range, as per standardization studies by Ferrario et al. (De Felício et al., 2009; Ferrario et al., 2000), is a condition of muscular steadiness of dental occlusion. A graphic representation of the difference between sEMG amplitude during MVC on cotton rolls and teeth was also achieved.

Statistical procedure provided that Kolmogorov–Smirnov test for normal distribution of observations was performed. Then, a parametric test (i.e., *t* test or analysis of variance, when needed) was used to compare the functional indexes between patients with and without the various occlusal features.

The null hypothesis was that there are no differences in muscle function indexes with respect to the dento‐skeletal features. Statistical significance was set at *p* < 0.05. All statistical procedures were performed with the software SPSS 21.0 (IBM, Milan, Italy).

## 3 RESULTS

Gender differences were not significant in any of the above indexes (*p* > 0.05). Findings showed the absence of significant differences in any muscle index between subjects with and without the various dental features (*p* > 0.05).

Some tendencies were observed. For instance, the average POC of subjects with incisor midline deviation was low (~74%; Table 1). In addition, subjects with deep bite have negative ACT values that are higher, in terms of absolute value, than the positive value of subjects without deep bite, thus indicating a higher activation of the temporalis muscles (Table 2).

**Table 1:** Mean and *SD* values of indexes of muscle function in subjects with and without midline deviation

	Subjects with incisor midline deviation	Subjects without incisor midline deviation
Number	11	24
POC	74.9 ± 15.57	82.2 ± 2.00
IMP	1.01 ± 0.39	1.02 ± 0.29
ASY	3.40 ± 26.83	−3.14 ± 7.99
ACT	−1.62 ± 18.68	0.98 ± 10.47
TC	−1.3 ± 12.51	−0.48 ± 7.13

*Note*. POC: percentage overlapping coefficient; IMP: impact; ASY: asymmetry; ACT: activation; TC: torque.

**Table 2:** Mean and *SD* values of indexes of muscle function in subjects with and without deep bite

	Subjects with deep bite	Subjects without deep bite
Number	8	27
POC	82.47 ± 2.20	79.17 ± 10.41
IMP	1.04 ± 0.29	1.01 ± 0.33
ASY	−6.66 ± 7.13	−0.56 ± 17.88
ACT	−4.82 ± 10.23	−0.11 ± 14.06
TC	−0.96 ± 4.46	−0.67 ± 10.00

*Note*. POC: percentage overlapping coefficient; IMP: impact; ASY: asymmetry; ACT: activation; TC: torque.

Any significant difference was found between muscle indexes of subjects with and without crossbite (Table 3).

**Table 3:** Mean and *SD* values of indexes of muscle function in subjects with and without crossbite

	Subjects with crossbite	Subjects without crossbite
Number	3	32
POC	81.02 ± 4.47	79.81 ± 9.63
IMP	0.78 ± 0.48	1.043 ± 0.30
ASY	6.92 ± 10.80	−1.8384 ± 16.60
ACT	2.14 ± 3.66	−1.49 ± 13.85
TC	0.88 ± 7.24	−0.8916 ± 9.20

*Note*. POC: percentage overlapping coefficient; IMP: impact; ASY: asymmetry; ACT: activation; TC: torque.

## 4 DISCUSSION

Historically, clinicians and researchers of several medical fields have been intrigued by the relationship between form and function. In dentistry, current knowledge suggests that contrarily to past beliefs, dental occlusion can have biological variations that should be considered physiological (i.e., not associated with signs and symptoms of dysfunction) independent on their specific configuration. Studies on the lack of association between dental occlusion and TMDs have thus diminished the etiological role of interarch relationships (Manfredini et al., 2012; Manfredini, Lombardo, & Siciliani, 2017a; Manfredini, Lombardo, & Siciliani, 2017b; Manfredini, Peretta, Guarda‐ Nardini, & Ferronato, 2010; Manfredini, Perinetti, & Guarda‐Nardini, March 2014; Manfredini, Perinetti, Stellini, Di Leonardo, & Guarda‐Nardini, 2015). Nonetheless, a full comprehension of the interaction between the different components of the stomatognathic system is far from achieved.

This study aimed to refine findings of previous studies on jaw muscle function with respect to different dental features. Results support a preliminary suggestion by Ferrario et al., who did not retrieve any differences in muscle function features between individuals with molar class subdivisions (Ferrario et al., 2006). The slight differences here reported in muscle function indexes of TMD‐free individuals with and without specific dental features are not significant. Thus, it can be confirmed that stomatognathic function is the result of a balanced equilibrium between the different structures, and it does not necessarily lie in a peculiar and ideal configuration (Ferrario et al., 2000; Okeson, 1985). As a consequence, all sEMG parameters, even of subjects with different dental morphology, can be expressed as a range of biological variability, instead of ideality (Ferrario, Sforza, & Serrao, 1999; Moreno et al., 2008; Serrao, Sforza, Dellavia, Antinori, & Ferrario, 2003). Thus, despite it is quite obvious that form and function influence each other reciprocally (Ferrario et al., 1999; Moss, 1969), the actual physiology of jaw muscles function with respect to facial and dental form is yet to clarify (Manfredini et al., 2013).

The literature on the topic is very poor (Ferrario et al., 2006), and the present investigation did not allow identifying physiological parameters, not even a range, proper of each subsample of individuals featuring specific dental morphologies. Future studies should be performed on enlarged samples, which allow an assessment of function‐form relationship within a multiple variable framework that better resembles the stomatognathic system. In addition, an inclusion of the evaluation of skeletal features by means of cephalometry is recommended. Notwithstanding, it seems plausible that investigating sEMG activity of jaw muscles in terms of differential activity of jaw muscles, with respect to each other, the two sides, and the different force task, is the most suitable strategy to get deeper into the issue of muscle functioning in different facial and occlusal types (De Felicio, Sidequersky, Tartaglia, & Sforza, 2009; Ferrario et al., 2000; Ferrario et al., 2006).

For instance, because the activation index gives information on the antero‐posterior balance, it is plausible that negative values in individuals with deep bite is due to a predominant temporalis activity to compensate for the twofold anterior occlusal planes in the presence of a high number of anterior contacts. Similarly, individuals with open bite should also have a negative ACT to compensate for the twofold posterior occlusal planes and find a better equilibrium between the dental arches. In support of this concept, Moreno et al. (2008) found a higher temporalis activation in subjects with Class II (Bakke, 1993). Another study found out an altered muscles coordination in healthy subjects with monolateral crossbite compared with subjects with normal occlusion during mastication on the side of the crossbite (Ferrario et al., 1999). Moreover, using this protocol, Ferrario et al. showed significant differences between sEMG activity of jaw muscles in healthy subjects with different vertical dimension, because subjects with a reduced vertical dimension showed higher potentials than long face subjects (Manfredini et al., 2010). Such findings supported other investigations that reported a negative correlation between muscles activity and long face types (Tecco, Crincoli, Di Bisceglie, Caputi, & Festa, 2009; Ueda, Ishizuka, Miyamoto, Morimoto, & Tanne, 1998).

The peculiarity of the above strategy to normalize EMG signal (i.e., MVC on cotton rolls, with the same electrodes, cables, and EMG apparatus, and on the same skin area) seems the most reasonable approach to reduce all biological and technical noises (Ferrario et al., 2000). Nonetheless, the fact that no differences have been here detected between muscle function of individuals with and without certain occlusal features suggests that the search for physiological parameters that explain the form‐function relationship might even reveal an unrealistic expectation. Future investigations combining multiple dental and skeletal features in the attempt to individualize parameters of muscle physiology are thus recommended.

## 5 CONCLUSIONS

The present investigation compared some indexes of muscle activity in healthy individuals with and without specific occlusal features, to assess the possible relationship between form and function and to try establishing some parameters of normality. Despite some minor differences were pointed out, none of them was significant. Thus, the interaction between form and function is too complex for hypothesizing a simple one‐to‐one relationship between interarch tooth relationship and muscle function patterns. Even if future researches on the topic are recommended by the use of multiple variable models, it is realistic to hypothesize that the physiology of jaw muscles has such a wide range of biological variation that correct function cannot be resembled in any specific configuration.

## CONFLICT OF INTEREST

The authors declare that they do not have any conflicts of interest concerning this manuscript.

## FUNDING INFORMATION

The authors declare they did not receive any funding for this investigation.
